# Synergistic Enhancement of Oxygen Permeability in Silane-Modified Hydrogel Networks for Advanced Ophthalmic Applications

**DOI:** 10.3390/gels11120987

**Published:** 2025-12-08

**Authors:** Min-Jae Lee, A-Young Sung

**Affiliations:** 1 Department of Optometry, Jeju Tourism University, Jeju 63063, Republic of Korea; mjlee@jtu.ac.kr; 2 Department of Optometry & Vision Science, Daegu Catholic University, Gyeongsan 38430, Republic of Korea

**Keywords:** silicone monomer, acrylic monomer, silane group, contact lens material, physical property

## Abstract

This study investigates the compatibility of various acrylic and silane monomers and aims to develop a high-performance hydrogel ophthalmic polymer. The formulations incorporated 2-(trimethylsiloxy)ethyl methacrylate (2TSEMA), 3-(methacryloxy)propyl tris(trimethylsiloxy)silane (3TRIS), and (1,1-dimethyl-2-propyl)oxy-trimethylsilane (TRIS) as functional additives to a base composition of silanol-terminated silicone (Sil-OH), N,N–dimethyl acrylamide (DMA), methyl methacrylate (MMA), and methyl acrylate (MA). Copolymerization was carried out using ethylene glycol dimethacrylate (EGDMA) as the crosslinking agent and azobisisobutyronitrile (AIBN) as the thermal initiator. All synthesized hydrogel lenses exhibited excellent optical transparency, indicating good monomer compatibility. The optical and physicochemical properties of the hydrogels varied depending on monomer composition. Notably, the formulation combining 2TSEMA with 1 wt% TRIS showed enhanced oxygen permeability, suggesting a synergistic interaction between the two silane-based components. These results demonstrate the potential of such hybrid formulations for use in next-generation functional hydrogel ophthalmic lenses.

## 1. Introduction

Hydrogels are three-dimensional crosslinked polymeric networks capable of absorbing and retaining large quantities of water while maintaining mechanical integrity. Their unique ability to combine softness, high oxygen permeability, and excellent biocompatibility has led to wide adoption in ophthalmic, biomedical, and environmental applications [1,2,3,4,5,6,7]. In particular, hydrogel-based contact lenses represent one of the most mature and clinically successful hydrogel technologies. The hydrated polymer matrix enables high oxygen transport rates, preventing corneal hypoxia and ensuring long-term ocular comfort [2,3]. The water-rich, elastic nature of these materials also allows surface wettability improvement, providing superior lubrication and minimal mechanical irritation to the ocular epithelium [4,5]. Moreover, recent developments in silicone-hydrogel systems combine the gas permeability of siloxane segments with the hydrophilicity of traditional hydrogels, enabling extended wear and enhanced tear exchange [6,7]. Consequently, hydrogels have become indispensable materials for contact lenses, wound dressings, tissue scaffolds, and drug delivery systems, owing to their biocompatibility, tunable mechanical properties, and controllable water content. A unique advantage of hydrogels lies in their tunability. Their physicochemical properties can be systematically adjusted by molecular design. By modifying monomer composition, crosslinking density, or network hydrophilicity, researchers can fine tune elasticity, transparency, permeability, and surface energy. The incorporation of secondary structures such as nanoparticles, block copolymers, or nanofillers has been shown to reinforce mechanical integrity and influence swelling dynamics [8,9]. These design strategies have enabled hydrogel lenses to evolve beyond vision correction toward more advanced functions, including sustained drug release, diagnostic sensing, and therapeutic treatment for ocular disorders [10].

Despite such progress, traditional hydrogels typically based on hydrophilic polymers such as poly(2-hydroxyethyl methacrylate) (pHEMA) suffer from a fundamental limitation: insufficient oxygen permeability. Because oxygen diffusion through water is relatively slow, conventional hydrogel lenses often fail to provide adequate oxygen transmission to the corneal epithelium during prolonged wear. This hypoxic environment can lead to complications such as epithelial edema, microcysts, or corneal neovascularization, which compromise both corneal physiology and visual acuity [11,12]. Consequently, the development of silicone based hydrogels has marked a pivotal advancement in the field.

Silicone hydrogels integrate oxygen permeable siloxane moieties, such as polydimethylsiloxane (PDMS) or tris(trimethylsiloxy)silylpropyl methacrylate (TRIS), into the polymer backbone. These Si–O–Si segments exhibit high free volume and low cohesive energy density, allowing rapid gas diffusion compared to aqueous domains. The resulting materials exhibit oxygen permeability (Dk) values several times greater than those of conventional hydrogels, effectively mitigating corneal hypoxia [13]. Furthermore, silicone hydrogels demonstrate enhanced mechanical robustness, improved tear resistance, and long-term shape retention properties essential for high performance and extended-wear contact lenses. However, this benefit comes at a cost: the intrinsic hydrophobicity of silicone leads to poor surface wettability and uneven tear film distribution, resulting in discomfort, protein deposition, and reduced biocompatibility during extended wear [7,14]. Addressing the delicate hydrophilic–hydrophobic balance in silicone-based systems remains a central challenge in hydrogel materials design.

In this context, organosilane chemistry offers a versatile strategy to bridge the gap between oxygen permeability and surface hydrophilicity. Silanes—organosilicon compounds in which a central silicon atom is covalently bonded to four substituents—display inherently high oxygen permeability due to the flexibility of the Si–O backbone and its low intermolecular cohesive energy. Unlike pure silicone, silane derivatives can be engineered to carry both hydrophobic and hydrophilic functional groups, enabling control over miscibility and interfacial interactions within polymer matrices. The chemical versatility of silanes allows them to participate in radical polymerization reactions, hydrogen bonding, and even coordination with metal ions, yielding hybrid organic–inorganic architectures with diverse properties [15,16,17].

Recent studies have explored silane coupling reactions to functionalize hydrogel matrices, leading to improved crosslink uniformity and enhanced surface energy control. For instance, Réthoré et al. reported the successful silanization of chitosan-based hydrogels, demonstrating enhanced mechanical performance and bioactivity in scaffold systems [18]. Such chemical adaptability highlights the potential of silane modification to engineer multifunctional polymeric networks for biomedical use.

The potential of silane-functionalized polymers extends well beyond ophthalmic materials. In microelectronics, silane-based coatings are used to form protective, gas-permeable barriers; in catalysis and sensing, silane linkers stabilize metal oxide nanostructures; and in biomedical engineering, silane coupling agents improve cell material interactions through tailored surface chemistry. However, despite these extensive applications, the incorporation of silane monomers into soft, transparent, and biocompatible hydrogel matrices—particularly for ophthalmic use—remains underexplored. Few studies have systematically examined how silane molecular geometry, chain length, and substitution patterns affect polymer morphology, crosslink distribution, and gas transport pathways in hydrated environments. Recently, Tran, N.P.D. et al. reported that nanocomposite silicone hydrogels enhanced with silane functionalized nanoparticles achieved unprecedented oxygen permeability while maintaining optical clarity [19], reinforcing the potential of silane chemistry in ophthalmic biomaterials.

The present study addresses this knowledge gap by investigating silane-modified silicone hydrogel systems for advanced ophthalmic applications. Specifically, we focus on the copolymerization of 2-(trimethylsiloxy)ethyl methacrylate (2TSEMA) and tris(trimethylsiloxy)silylpropyl methacrylate (TRIS) with conventional methacrylate monomers to create siloxane enriched networks. These two monomers were selected due to their complementary characteristics: 2TSEMA provides moderate hydrophilicity and flexibility, whereas TRIS contributes high gas permeability and mechanical strength through its bulky siloxane substituents. By combining these components, the study aims to achieve synergistic improvements in oxygen diffusion and hydration stability while preserving optical transparency and mechanical uniformity.

To achieve these objectives, the work follows a rational materials design framework comprising synthesis, characterization, and structure property correlation. First, a series of hydrogel formulations containing varying proportions of silane monomers were synthesized via free-radical copolymerization, using ethylene glycol dimethacrylate (EGDMA) as a crosslinker and azobisisobutyronitrile (AIBN) as a thermal initiator. The resulting hydrogels were purified, hydrated, and subjected to systematic physicochemical characterization. Oxygen permeability was measured using the polarographic method in compliance with ISO 18369-4:2017 [20], while surface wettability was evaluated through contact angle and atomic force microscopy (AFM) analyses. Additionally, optical transmittance, refractive index, water content, and thermal stability were quantified to assess correlations between silane incorporation and network performance.

This study focuses on understanding how silane incorporation affects the oxygen permeability and hydration balance of silicone hydrogel contact lenses. By optimizing silane loading, the materials achieved enhanced oxygen diffusion without compromising transparency or surface wettability.

The implications of this work are primarily confined to ophthalmic silicone-hydrogel applications, particularly in improving oxygen transport, mechanical robustness, and long-term shape stability of soft lenses. Although the silane-modification approach could, in principle, be extended to other biomedical hydrogels, such applications would require further evaluation of their tear resistance, dimensional stability, and biocompatibility in physiological environments [21,22,23].

## 2. Results and Discussion

### 2.1. Polymerization Stability

#### 2.1.1. Thermal Analysis

Thermogravimetric analysis (TGA) was carried out under nitrogen atmosphere at a heating rate of 10 °C/min to examine the thermal resilience and decomposition behavior of the silane-modified networks. In the 2TT1 sample, the onset of thermal degradation was observed at approximately 374.05 °C, with the major decomposition peak centered at 415.68 °C. At 326.69 °C, about 90% of the initial mass remained, and by 370.62 °C, 80% remained. The 3TT1 sample exhibited a slightly higher onset point of 375.63 °C and a peak at 414.47 °C; at 352.71 °C, 90% mass retention was noted, and at 379.37 °C, 80% remained. These data suggest that both modified hydrogels maintain strong thermal stability well above 300 °C, with primary decomposition peaks near 415 °C.

The thermal degradation profiles implies that integration of silane and acrylic monomers occurred without large-scale phase separation, pointing toward a homogeneous copolymer network. Comparable studies on polymer–silane composites have reported analogous thermal stability trends: for example, previous researchers demonstrated that silane-modified silicone resins retain high decomposition onset temperatures compared to parent silicones, affirming the thermal robustness of silicon–silane hybrid systems [24,25]. Moreover, silane coupling agents are known to contribute favorable thermal stability within polymer networks due to the strength of siloxane bonds and lower intermolecular cohesive energy density, which helps resist premature chain scission [26].

**Figure 1:**
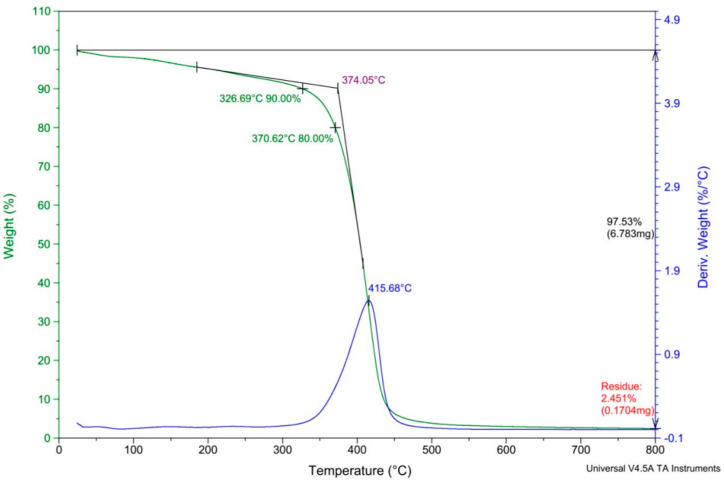
Typical TGA thermogram analysis of 2TT1 (upper curve) and 3TT1 (lower curve) sample.

Taken together, these TGA results confirm that our silane containing hydrogels possess solid thermal resilience suitable for ophthalmic and biomedical processing conditions, and that the structural integrity of the network remains intact until relatively high temperatures. The TGA results are shown in Figure 1.

#### 2.1.2. pH and Potassium Permanganate Reducing Analysis

**Figure 2:**
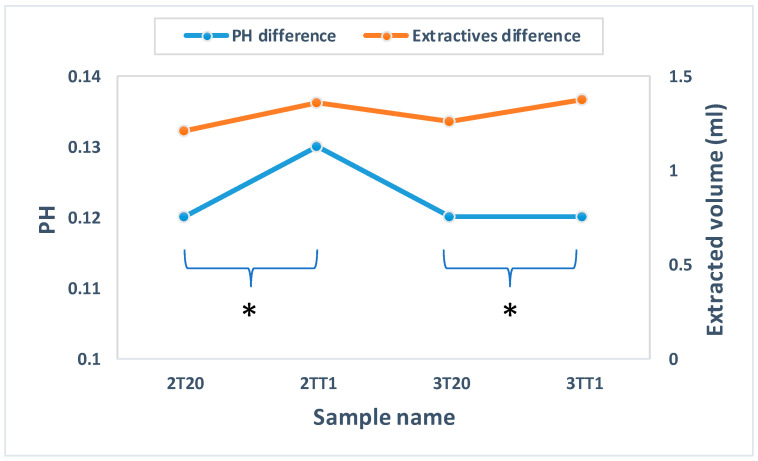
Extractables test of samples. (*: *p* < 0.05).

Polymerization stability was further interrogated by extractable testing using two orthogonal readouts: (i) the pH shift of lens eluates relative to the blank medium and (ii) the potassium-permanganate-reducing substance (KMnO_4_) assay, which is sensitive to oxidizable organic leachates such as residual monomers, initiator fragments, and low-molar-mass additives. Eluates were prepared under conditions aligned with contact lens physicochemical test standards and medical device chemical characterization guidance [26,27]. In our study, extracts from 2T20, 2TT1, 3T20, and 3TT1 were compared to assess whether 2TSEMA, 3TRIS, and TRIS leached from the matrix. The measured pH differences were 0.12, 0.13, 0.12, and 0.12, respectively—well below the ≤1.5-unit acceptance threshold commonly applied in hydrogel lens evaluations, indicating negligible acid/base-active extractables [27,28]. Likewise, the KMnO_4_ reducing volumes differed from the control by 1.21 ± 0.08 mL (2T20), 1.36 ± 0.13 mL (2TT1), 1.26 ± 0.43 mL (3T20), and 1.38 ± 0.51 mL (3TT1), all below the ≤2 mL criterion frequently used to denote “no detectable extractables” in ophthalmic hydrogel lenses [27,28]. Together, these findings demonstrate that incorporation of TRIS produced, at most, a marginal increase in oxidizable residues that did not reach methodological significance, consistent with substantially complete conversion and a low level of leachable organics. When interpreted alongside the thermal data, the minimal pH drift and low permanganate consumption support a picture of uniform copolymerization and chemical stability of the silane modified networks—key prerequisites for ocular safety and regulatory acceptance of new lens materials [26,27]. The summarized pH and KMnO_4_ results are shown in Figure 2.

### 2.2. Physical Properties

#### 2.2.1. Optical Transmittance

**Figure 3:**
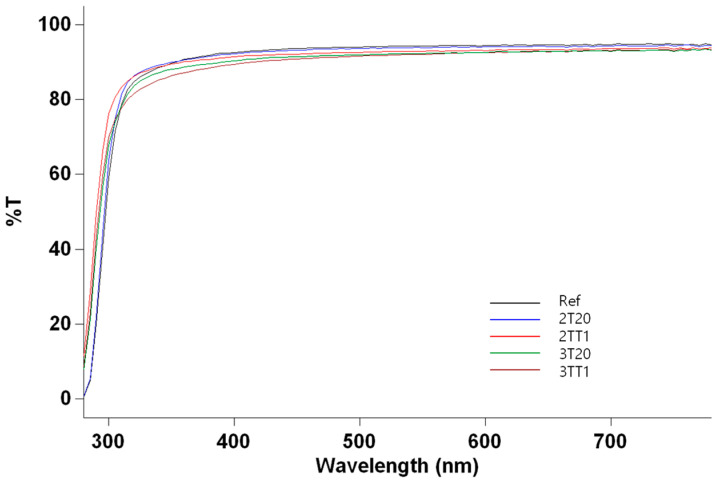
Optical transmittance distribution of samples.

The optical transmittance of all hydrogel formulations was evaluated in the UV-B (280–315 nm), UV-A (315–380 nm), and visible (380–780 nm) regions to assess their suitability for ophthalmic use. As shown in Figure 3, sample 2T20 exhibited transmittance values of 45.68% (UV-B), 84.60% (UV-A), and 91.44% (visible). Upon the incorporation of 1 wt% TRIS, the 2TT1 formulation showed slightly increased transmittance of 48.64% (UV-B), 85.29% (UV-A), and 90.38% (visible). Similarly, 3T20 displayed 42.93% (UV-B), 86.29% (UV-A), and 92.33% (visible), while its TRIS-modified counterpart 3TT1 yielded 42.92% (UV-B), 84.04% (UV-A), and 90.85% (visible).

All silane-modified hydrogels maintained optical transparency above 90% in the visible range, comparable to the optical clarity reported for commercial silicone hydrogel lenses [29]. The inclusion of TRIS did not cause any measurable spectral distortion or scattering, implying that silane incorporation did not disrupt polymer homogeneity or generate light absorbing microdomains. Similar trends have been observed by Tran et al., who reported that silicone hydrogel lenses synthesized from polydimethylsiloxane (PDMS) and hydrophilic monomers exhibited transmittance values exceeding 95% across the visible spectrum, even at elevated silicone content [29].

In addition, Wuchte et al. demonstrated that novel silicone macromer-based hydrogel lenses exhibit consistent light transmittance and minimized absorbance in the UV-visible range, confirming that careful copolymer design can maintain optical clarity while enhancing mechanical and oxygen transport properties [30]. The high visible transmittance and uniform spectra observed in this study therefore verify that the silane-modified hydrogels retained excellent transparency suitable for extended wear contact lens applications. The transmittance spectra are presented in Figure 3.

#### 2.2.2. Refractive Index and Water Content

The refractive index (n) and equilibrium water content (WEC) of the thermally polymerized samples were measured to probe how silane incorporation modulates the optical and hydration properties of the hydrogel network. In the reference (Ref.) sample lacking any silane additive, the refractive index was found to be 1.388 ± 0.01. Upon introduction of silane monomers, the refractive indices increased to 1.391 ± 0.00 (2T20) and 1.395 ± 0.01 (2TT1). For the 3T20 and 3TT1 samples, the measured refractive indices were both 1.415 ± 0.01. One-way ANOVA analysis confirmed that all silane-modified groups exhibited significantly higher refractive indices than the reference sample (*p* < 0.01), whereas no significant difference was observed between 3T20 and 3TT1 (*p* > 0.05). Concurrently, the EWC values decreased from 64.80 ± 0.89% (Ref) to 62.55 ± 1.49% (2T20), 62.53 ± 1.05% (2TT1), 52.77 ± 1.43% (3T20), and 50.39 ± 1.25% (3TT1). Statistical comparison using the same test indicated that the reduction in EWC was significant for all silane-containing formulations relative to the reference (*p* < 0.01). No significant difference was detected between 2T20 and 2TT1.

This inverse correlation between refractive index and equilibrium water content where increased silane loading leads to lower hydration but higher refractive index is consistent with established structure property trends for hydrogel lenses. Specifically, González-Méi-jome et al. demonstrated a strong linear correlation (R^2^ ≈ 0.979) between measured refractive index and equilibrium water content across both conventional and silicone hydrogels, enabling predictive models for one property based on the other [31]. Their work underscores that as water content falls, the polymer matrix densifies and polarizable volume per unit path length increases, driving refractive index upward.

Furthermore, some studies proposed polynomial relationships mapping sucrose-based hydration models to refractive index, reinforcing that such n–EWC interplay is quantifiable and applicable across lens materials [32,33]. It is noteworthy that, unlike conventional hydrogel systems where increasing water content tends to enhance comfort but reduce optical precision, the silane-modified silicone hydrogels in this study maintain high transparency and surface homogeneity even as EWC decreases. The optical clarity of all silane-based samples exceeded 90% transmittance in the visible range, confirming that the observed refractive changes originate from intrinsic material properties rather than morphological defects.

**Figure 4:**
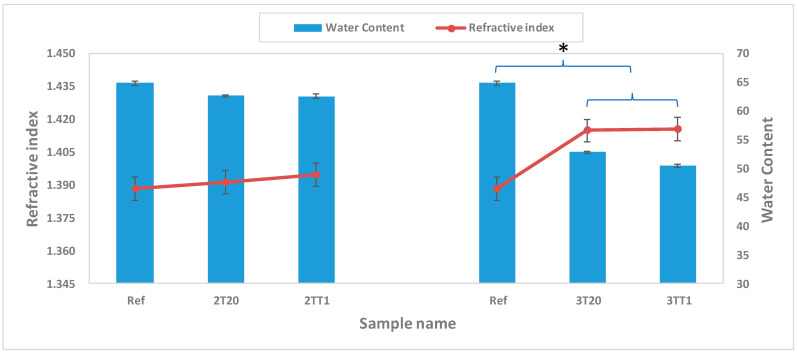
Refractive index and water content of samples. (*: *p* < 0.01).

Therefore, silane loading provides a reliable and predictable lever to fine tune refractive index while controlling hydration. This is particularly advantageous for lens design: adjusting silane concentration allows modulation of the optical power or base curve without compromising wearer comfort or physiological compatibility. The 3-series lenses (3T20, 3TT1) exemplify this trend, showing modest refractive increases with moderate water reduction, while the 2-series (especially 2TT1) exhibits more pronounced changes due to stronger siloxane network formation. These findings demonstrate that by manipulating silane composition, it is possible to achieve lenses with tailored refractive properties and hydration balance suited for diverse ophthalmic applications. The quantitative correlation between n and EWC is graphically depicted in Figure 4.

#### 2.2.3. Contact Angle and Water Content

Surface wettability plays a critical role in determining comfort, tear film stability, and deposit resistance in contact lens materials. To evaluate surface hydrophilicity, the static sessile drop contact angle was measured on hydrated lens surfaces. The reference (Ref.) hydrogel exhibited a high contact angle of 92.25 ± 1.56°, indicating poor wettability. Upon copolymerization with silane containing monomers, the contact angle significantly decreased to 62.55 ± 1.29° for 2T20 and 62.53 ± 1.99° for 2TT1, while 3T20 and 3TT1 exhibited even lower angles of 52.77 ± 2.79° and 50.39 ± 2.28°, respectively. Statistical analysis using one-way ANOVA confirmed that all modified samples showed significantly lower contact angles compared to the reference lens (*p* < 0.01). No significant difference was found between 2T20 and 2TT1 (*p* > 0.05). These results demonstrate that the incorporation of 2TSEMA and 3TRIS substantially improves surface hydrophilicity, promoting better tear film spreading and lubrication.

To further investigate the microstructural basis of this improvement, atomic force microscopy (AFM) was used to examine the nanoscale surface morphology. The arithmetic mean roughness (Ra) values decreased from 13.05 ± 1.52 nm for Ref to 9.42 ± 2.32 nm for 2TT1 and 3.02 ± 2.76 nm for 3TT1, confirming that silane incorporation produces smoother and more homogeneous surfaces. Statistical analysis using one-way ANOVA demonstrated a significant reduction in surface roughness for both silane-modified samples compared with the reference lens (*p* < 0.01). This enhancement in surface energy is attributed to increased distribution of polar functional groups at the polymer–water interface and a more uniform nanoscale morphology, both of which contribute to improved tear-film compatibility and reduced surface fouling [34,35].

Such behavior is consistent with prior reports by Eftimov et al., who observed that smoother, more hydrophilic silicone hydrogel surfaces reduce contact angle hysteresis and friction, thereby enhancing on eye comfort [35]. Similarly, Lin et al. demonstrated that the grafting of polyelectrolyte multilayers onto silicone hydrogel substrates led to progressive decreases in contact angle and improved wettability due to altered nanoscale roughness and enhanced hydration layer formation [36]. The measured static contact angles showed only marginal differences among samples. These small variations (<5%) fall within the experimental uncertainty of the measurement. The minor decrease in the 3T-series after TRIS addition may reflect subtle rearrangement of siloxane segments at the air–polymer interface, exposing slightly more polar groups. In contrast, the 2T-series maintains stable wettability because of the methacryloxypropyl group in 2TSEMA, which improves interfacial compatibility with the hydrophilic matrix and prevents surface segregation [7,29]. Overall, both systems exhibited excellent hydrophilic surface characteristics suitable for ophthalmic applications.

**Figure 5:**
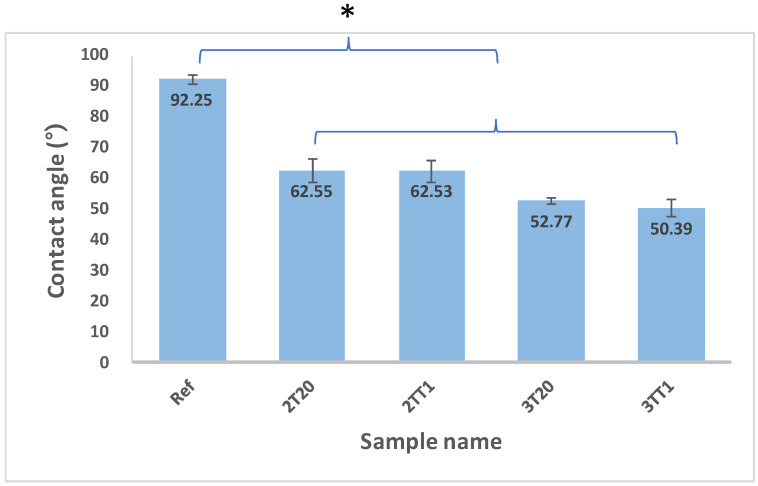
Contact angle of samples. (*: *p* < 0.01).

**Figure 6:**
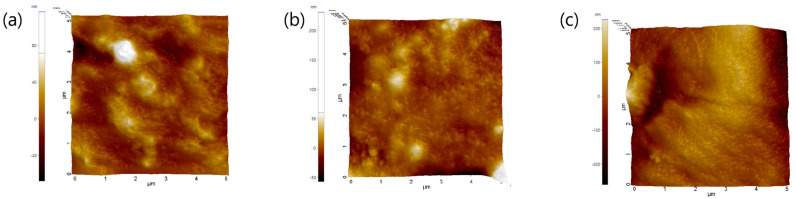
AFM image of lens samples for surface analysis. (**a**) Ref, (**b**) 2TT1, (**c**) 3TT1.

In the present work, the combined reduction in contact angle and surface roughness confirms that silane-modified hydrogels achieve synergistic improvements in both morphology and interfacial chemistry. These enhancements are expected to translate into superior tear film stability, reduced protein/lipid deposition, and extended wearing comfort, making the 3TT1 formulation particularly promising for next-generation ophthalmic applications. The contact angle data are shown in Figure 5 and AFM images are presented in Figure 6.

#### 2.2.4. Oxygen Permeability and Water Content

Oxygen permeability (Dk) is a fundamental property determining the physiological compatibility of contact lens materials, as insufficient oxygen transmission can cause corneal hypoxia, edema, and neovascularization. In this study, the reference hydrogel exhibited a baseline Dk of 25.79 ± 1.29 × 10^−11^ cm^2^ s^−1^ (mL O_2_/mL mmHg), while 2T20 and 2TT1 achieved markedly higher values of 30.53 ± 2.47 × 10^−11^ and 39.52 ± 3.40 × 10^−11^, respectively. Similarly, 3T20 and 3TT1 displayed 26.13 ± 1.32 × 10^−11^ and 30.73 ± 2.89 × 10^−11^, respectively. Statistical analysis using one-way ANOVA confirmed that all silane-containing formulations exhibited significantly higher Dk values than the reference hydrogel (*p* < 0.01). The most pronounced enhancement was observed in the 2TT1 formulation, corresponding to a nearly 50% increase in oxygen permeability compared to the reference. Importantly, these improvements were achieved despite a measurable decrease in water content, implying that the diffusion of oxygen occurs predominantly through the siloxane-rich phase rather than the aqueous phase.

This inverse relationship between Dk and water content is well documented in silicone hydrogel systems, where the primary diffusion pathway for oxygen arises from interconnected siloxane networks rather than water-filled pores [37]. Wu et al. demonstrated that silicone hydrogels exhibit morphology-dependent O_2_ transport governed by the degree of siloxane domain connectivity [38]. Similarly, Zhao et al. found that oxygen permeability strongly correlates with microphase separation morphology rather than overall hydration level [39]. Such behavior underscores the synergistic role of 2TSEMA and TRIS in forming interconnected siloxane domains that facilitate efficient O_2_ transport [37,38,39].

With three experimentally accessible benchmarks—Ref = 25.79, 2T20 = 30.53, and 2TT1 = 39.52 barrer—we evaluated synergy using a conservative (lower-bound) model because a TRIS-only lens could not be fabricated under identical conditions. The co-modified hydrogel attained 39.52 barrer, yielding an excess over additivity of 8.99 barrer. This corresponds to 34.86% of the reference Dk and accounts for 65.48% of the total improvement from Ref to 2TT1 (Table 1). Mechanistically, we attribute this to TRIS-derived siloxane diffusion microdomains that cooperate with 2T-induced polarity/free-volume increases, producing a co-continuous microstructure that reduces oxygen-path tortuosity.

**Figure 7:**
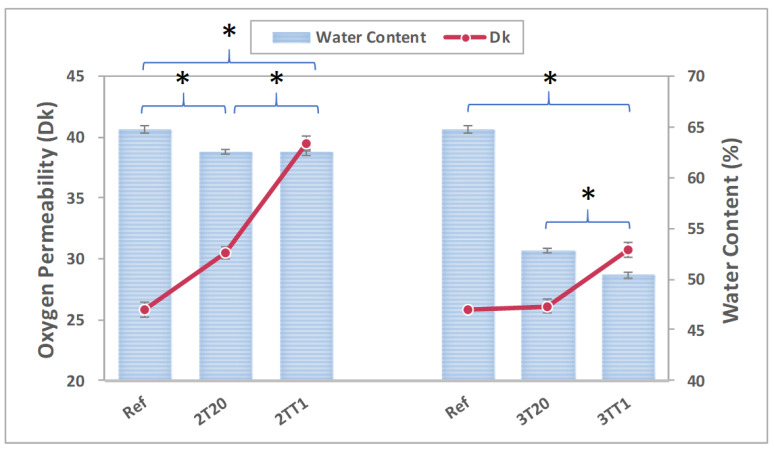
DK and water content of samples. (*: *p* < 0.01).

Collectively, these findings suggest that silane monomers enable molecular-level control over the continuity of oxygen conductive pathways within the hydrogel matrix. As a result, the lenses exhibited stable and efficient oxygen transport even at reduced hydration levels, ensuring the physiological integrity of the cornea and long-term ocular comfort. The correlation between oxygen permeability and water content is illustrated in Figure 7.

#### 2.2.5. Tensile Strength

**Figure 8:**
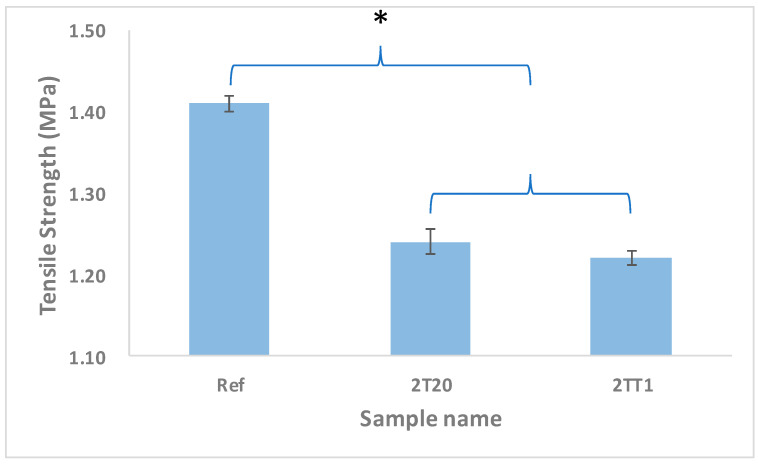
Tensile strength of samples. (*: *p* < 0.01).

The mechanical performance of the synthesized silicone hydrogels was evaluated by tensile testing. The tensile strength of Ref was 1.41 ± 0.12 MPa, that of 2T20 was 1.24 ± 0.15 MPa, and that of 2TT1 was 1.21 ± 0.10 MPa. All samples exhibited mechanical robustness comparable to commercial silicone hydrogel lenses, maintaining adequate strength for handling and forming while preserving flexibility [30]. The results indicate that the incorporation of TRIS and 2TSEMA slightly decreased tensile strength due to increased siloxane content, yet the overall mechanical stability remained sufficient for practical ophthalmic use [40]. A graph of the tensile strength of each sample is shown in Figure 8.

### 2.3. Limitations

In this study, although indirect analyses such as extractable content, pH stability, and optical clarity were performed to verify the successful polymerization and purity of the hydrogels, direct spectroscopic characterization (e.g., FTIR or NMR) was not conducted due to equipment limitations. Future studies will incorporate these techniques to further confirm the chemical composition and crosslinked structure of the synthesized silicone hydrogel network.

## 3. Conclusions

We have developed a series of silane-modified silicone hydrogel materials that exhibit enhanced oxygen permeability, high optical transparency, and superior structural stability, achieved through the synergistic interaction of 2TSEMA and TRIS monomers. The optimized formulation (2TT1) demonstrated up to a 50% increase in oxygen permeability without compromising mechanical strength or optical transparency, which is attributed to the formation of continuous siloxane-rich microdomains that facilitate efficient oxygen transport through the polymer matrix. These results confirm that controlled silane incorporation serves as an effective molecular design strategy for increasing the gas permeability, hydration balance, and surface wettability of hydrogel-based ophthalmic materials.

The silane-modified network not only offers a balance between hydrophobic and hydrophilic domains but also presents reactive sites that can be further functionalized for drug delivery or biosensing. Future studies will aim to integrate bioactive functionalities such as sustained drug release, antimicrobial activity, and adaptive surface hydration through the introduction of functionalized silanes or nanostructured fillers. Furthermore, optimization of silane composition and crosslink density could yield smart hydrogel systems with stimuli-responsive characteristics, suitable for next-generation biomedical applications including wound healing matrices, tissue engineering scaffolds, and ocular surface therapeutics.

Collectively, the incorporation of 2TSEMA and TRIS into silicone-based hydrogel networks resulted in a synergistic enhancement of oxygen permeability while maintaining hydration stability. These findings establish a robust foundation for the rational molecular engineering of silane-based hydrogels, providing a versatile platform for the design of advanced biomedical and ophthalmic devices that combine superior oxygen transport, optical clarity, chemical stability, and extended wearing comfort.

## 4. Materials and Methods

### 4.1. Reagents and Materials

For the fabrication of contact lenses, the silicone monomer (Sil-OH), N,N–dimethyl acrylamide (DMA), methyl methacrylate (MMA), and methyl acrylate (MA) were used. Ethylene glycol dimethacrylate (EGDMA) was used as a crosslinking agent, and azobisisobutyronitrile (AIBN) was used as an initiator. To impart functionality to the lenses, 2-(trimethyl siloxy)ethyl methacrylate (2TSEMA), 3-(methacryloxy)propyl tris(trimethylsiloxy)silane (3TRIS), and (1,1-dimethyl-2-propyl)oxy-trimethyl silane (TRIS) were added. Except for the synthesized silicone monomer, all monomers used were purchased from Sigma-Aldrich (St. Louis, MO, USA).

### 4.2. Polymerization

A series of silicone hydrogel formulations were synthesized via free-radical copolymerization of silanol-terminated silicone monomer (Sil–OH), methyl methacrylate (MMA), methyl acrylate (MA), and N,N-dimethylacrylamide (DMA). All monomers and reagents were of analytical grade and used as received: Sil–OH and 3-(methacryloxy)propyl tris(trimethylsiloxy)silane (3TRIS), MMA, MA, DMA, ethylene glycol dimethacrylate (EGDMA, 98%), and azobisisobutyronitrile (AIBN, 97%) were obtained from Sigma-Aldrich (St. Louis, MO, USA). Crosslinking was achieved using EGDMA, and polymerization was initiated by AIBN. To prepare the polymerization solution, the mixture of monomers was stirred at room temperature for 50 min using a vortex mixer (Vortex GENIE 2, Scientific Industries, Bohemia, NY, USA), followed by additional mixing for 30 min using an ultrasonic homogenizer to ensure complete dispersion. Free-radical copolymerization was then initiated by adding AIBN (0.3 wt%) as a thermal initiator. Upon heating, AIBN decomposed to generate free radicals, which triggered copolymerization among the monomers to form a crosslinked hydrogel network. To elucidate the effect of silane incorporation, 2TSEMA and 3TRIS were introduced at concentrations ranging from 10 to 30 wt%, followed by additional modification with 1 wt% TRIS, yielding optimized compositions designated 2TT1 and 3TT1. Each formulation was cast into a 0.00 D polypropylene mold supplied by Baeksan Co., Ltd. (Busan, Republic of Korea) and polymerized in a programmable convection oven at 85 °C for 1 h, followed by post-curing at 120 °C for 30 min. After polymerization, the hydrogel lenses were removed from the molds and soaked in ethanol for 24 h to remove unreacted monomers. They were then rinsed in distilled water for 1 h to eliminate residual ethanol and subsequently hydrated in 0.9% saline solution for 24 h prior to measuring and comparing their physical properties. Thermal curing under the described conditions produced transparent and uniform copolymerization and stable network formation. The detailed formulation ratios and sample designations are summarized in Table 2.

### 4.3. Experimental Method

The refractive index of the fabricated lenses was measured in a hydrated state using an ABBE Refractometer (ATAGO DR-A1, ATAGO Co., Ltd., Tokyo, Japan) in accordance with ISO 18369-4:2017. The water content was determined by the gravimetric method, also following ISO 18369-4:2017. Spectral transmittance was measured using a spectral transmittance meter (Agilent Cary 60 UV-Vis, Agilent, San Clara, CA, USA), and the results were classified into UV-B (280–315 nm), UV-A (315–380 nm), and visible light (380–780 nm) regions. Wettability was evaluated by measuring the contact angle using a contact angle instrument (Kruss GmbH, DSA30, Hamburg, Germany) based on the sessile drop method. Oxygen permeability was assessed using the polarographic method specified in ISO 18369-4:2017, Ophthalmic optics—Contact lenses—Part 4: Physicochemical properties of contact lens materials. To examine the lens surface morphology and roughness, atomic force microscopy (AFM, XE-100, Park Systems, Seoul, Republic of Korea) was employed. Images were acquired at a scan size of 5 µm × 5 µm with a scan rate of 0.3 Hz in non-contact mode. Three random areas were scanned per sample, and the image resolution was 256 × 256 pixels. To assess extractables, pH measurement and the potassium permanganate-reducing substance test were conducted. Thermal stability was analyzed using thermogravimetric analysis (TGA) with a TGA Q500 instrument (TA Instruments, New Castle, DE, USA), by observing weight changes according to decomposition temperature. Tensile strength was measured with a tensile test machine AGS-X 20N (SHIMADZU, Kyoto, Japan). When a force of 0 to 2.00 kgf was applied to both sides of the sample at a rate of 10 mm/1 min while the surface moisture of the sample was removed, the maximum damage to the lens was measured and analyzed as a tensile strength value. All measurements were performed in quintuplicate (*n* = 5).

## Figures and Tables

**Table 1:** Quantitative contribution of 2TSEMA and TRIS to oxygen permeability enhancement.

Sample Name	Dk (Barrer)	ΔDk (Barrer)	Additive Expectation (Barrer)	Excess Over Additive Expectation (Barrer)	Synergy (%)
Ref	25.79 ± 1.29				
2T20	30.53 ± 2.47	+4.74			
2TT1	39.52 ± 3.40	+13.73	30.53	8.99	34.86

**Table 2:** Percent composition of samples. (Unit: wt%).

	Sil-OH	DMA	MMA	MA	EGDMA	AIBN	2TSEMA *	3TRIS **	TRIS ***	Total
Ref	32.29	64.58	0.97	0.97	0.99	0.20	-	-	-	100
2T10	29.36	58.71	0.88	0.88	0.90	0.18	9.09	-	-	100
2T20	26.91	53.82	0.81	0.81	0.82	0.16	16.67	-	-	100
2T30	24.84	49.68	0.75	0.75	0.76	0.15	23.08	-	-	100
3T10	29.36	58.71	0.88	0.88	0.90	0.18	-	9.09	-	100
3T20	26.91	53.82	0.81	0.81	0.82	0.16	-	16.67	-	100
3T30	24.84	49.68	0.75	0.75	0.76	0.15	-	23.08	-	100
2TT1	26.64	53.29	0.80	0.80	0.82	0.16	16.50	-	0.99	100
3TT1	26.64	53.29	0.80	0.80	0.82	0.16		16.50	0.99	100

* 2TSEMA: 2-(trimethyl siloxy)ethyl methacrylate. ** 3TRIS: 3-(methacryloxy)propyl tris(trimethylsiloxy)silane *** TRIS: (1,1-dimethyl-2-propyl)oxy-trimethyl silane.

## Data Availability

The original contributions presented in this study are included in the article. Further inquiries can be directed to the corresponding author.
